# USP34 regulates tooth root morphogenesis by stabilizing NFIC

**DOI:** 10.1038/s41368-021-00114-8

**Published:** 2021-03-09

**Authors:** Shuang Jiang, Rui Sheng, Xingying Qi, Jun Wang, Yuchen Guo, Quan Yuan

**Affiliations:** 1 State Key Laboratory of Oral Diseases & National Clinical Research Center for Oral Diseases & West China Hospital of Stomatology, Sichuan University, Chengdu, China; 2 Department of Periodontology, West China Hospital of Stomatology, Sichuan University, Chengdu, China; 3 Department of Oral Surgery, West China Hospital of Stomatology, Sichuan University, Chengdu, China; 4 Department of Oral Implantology, West China Hospital of Stomatology, Sichuan University, Chengdu, China

**Keywords:** Differentiation, Mesenchymal stem cells

## Abstract

Tooth root morphogenesis involves two biological processes, root elongation and dentinogenesis, which are guaranteed by downgrowth of Hertwig’s epithelial root sheath (HERS) and normal odontoblast differentiation. Ubiquitin-dependent protein degradation has been reported to precisely regulate various physiological processes, while its role in tooth development is still elusive. Here we show ubiquitin-specific protease 34 (USP34) plays a pivotal role in root formation. Deletion of *Usp34* in dental mesenchymal cells leads to short root anomaly, characterized by truncated roots and thin root dentin. The *USP34*-deficient dental pulp cells (DPCs) exhibit decreased odontogenic differentiation with downregulation of nuclear factor I/C (NFIC). Overexpression of NFIC partially restores the impaired odontogenic potential of DPCs. These findings indicate that USP34-dependent deubiquitination is critical for root morphogenesis by stabilizing NFIC.

## Introduction

Formation of the tooth root enables the tooth anchor to alveolar bones via periodontal ligament, making it an indispensable process in tooth eruption and development of the lower face. Tooth roots also play a role in transmitting occlusal forces to the surrounding alveolar bones during mastication. Developmental defect of roots reduces alveolar bone support and thus perturbs oral function. Though dental prosthesis will to some extent restore appearance and oral function, it cannot achieve full, unrestricted maxillofacial function. Tooth tissue regeneration has been presented as a promising treatment.^1,2^ It has been demonstrated that separated dental mesenchyme and epithelium can form teeth when recombined and cultured.^3,4^ Therefore, elucidating the process of root morphogenesis and the complicated mechanisms involved is a prerequisite for rapidly and efficiently evaluating and developing oral clinical therapies.

Tooth root morphogenesis is a dynamic process in which dental mesenchymal cells migrate and differentiate into odontoblasts and dental pulp cells, shaping the future root of tooth. Interaction between the dental mesenchyme and epithelium is an important regulator of tooth development.^5,6^ Root formation is initiated after crown formation, marked by the occurrence of a bilayered tissue known as HERS.^7^ The roots earn their first shapes when the HERS begins to grow downward, and subsequently the adjacent dental papilla mesenchymal cells are induced and differentiate into odontoblasts to form dentin.^8,9^ Later interruption and perforation of HERS enables the outer dental follicle cells to contact newly formed dentin via the mesh-like structure. Disorders in any of the steps mentioned may disturb normal root development. Recent studies have shown that several signaling pathways participate in root morphogenesis, such as Wnt/β-catenin, transforming growth factor β (TGF-β), and sonic hedgehog (shh) signaling pathways.^10–12^ Conditional knocking out Wnt10a, Wls or inhibiting Wnt/β-catenin signaling through DKK1 affected cell proliferation and differentiation, and then disrupt dentin formation and root morphogenesis.^13–16^ Deficiency of Smad7, a general antagonist against TGF-β signaling, led to reduced tooth size and affected cell proliferation.^17^ The question that remains to be answered then, is when and how these molecules achieve their specificity during tooth development.

The ubiquitin-proteasome system, a key protein degradation system which marks protein with ubiquitin, has been implicated in various biological processes.^18^ Besides, deubiquitinases remove ubiquitin marks and stabilize target proteins to ensure the coordinated function of the whole system.^19^ So far, only two E3 ubiquitin ligases, Smurf1 and Mdm2, have been identified as regulatory factors in odontogenic differentiation.^20–22^ Despite extensive researches having been conducted into ubiquitination, the precise molecular network controlling deubiquitylation in tooth development is yet to be determined. Thus, there is an urgent need to clarify the effect of deubiquitination on tooth root morphogenesis.

Previously, we found that ubiquitin-specific protease 34 (USP34), a member of the largest family of deubiquitinases, controled bone homeostasis through regulating the function and differentiation of osteoblasts as well as osteoclasts.^23–25^ Herein we generated *Sp7-Cre;Usp34*^*fl/fl*^ mice to decipher its biological function in root morphogenesis and provided evidence that absence of USP34 in dental mesenchymal cells caused short root anomaly. Mechanically, we demonstrated that USP34 deubiquitinates and stabilizes NFIC during odontoblast differentiation.

## Results

### Expression of USP34 in dental pulp

**Fig. 1:**
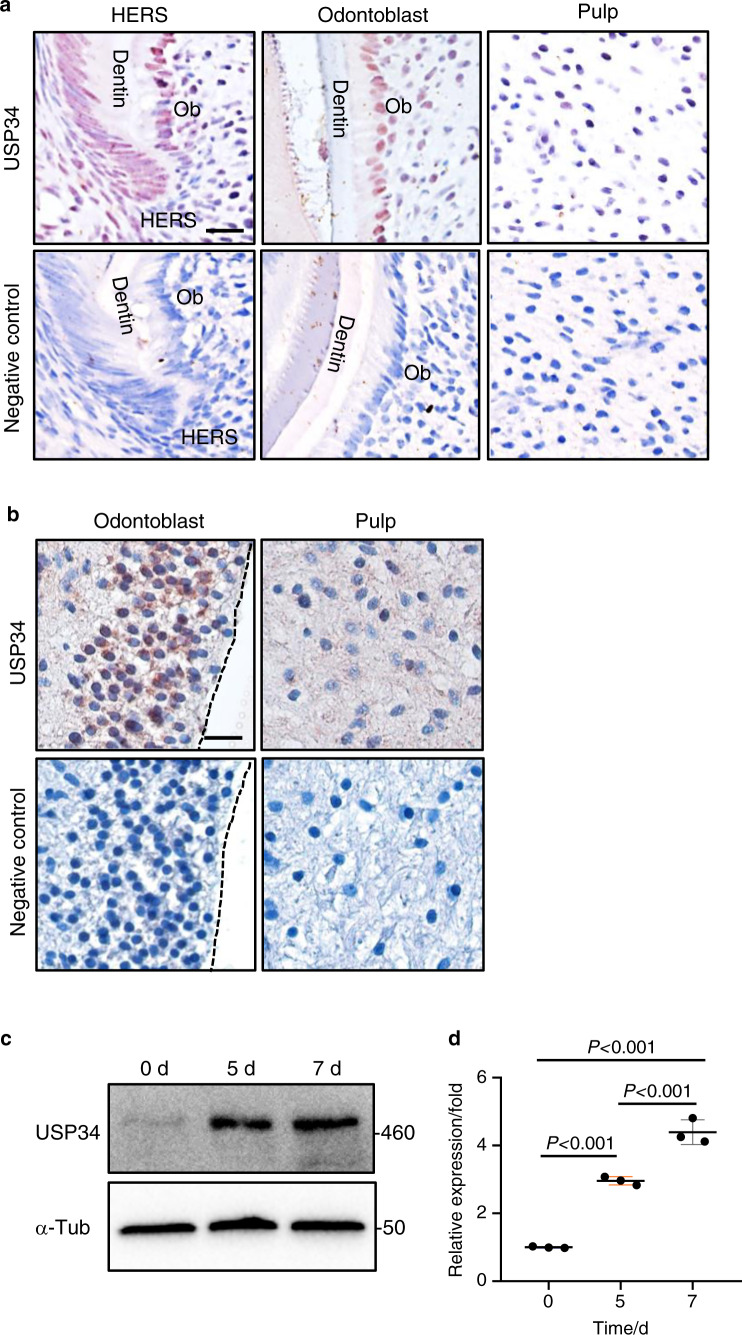
Expression of USP34. **a** Immunohistochemical staining of USP34 in mouse P7 molar. USP34 is expressed weakly in dental pulp, strongly in the HERS and the differentiated odontoblasts. Ob Odontoblasts. Scale bar: 10 μm. **b** Immunohistochemical staining of USP34 in dental pulp tissues from intact human teeth. USP34 is highly expressed in differentiated odontoblasts. Scale bar: 10 μm. **c** Western blot analysis of USP34 in the DPCs cultured in odontogenic medium. **d** mRNA level of *USP34* in DPCs cultured in odontogenic medium (*n* = 3)

To identify the potential role of USP34 in tooth root formation, we first performed immunohistochemistric staining of USP34 in the mouse molars sections, and noticed that it was highly expressed in the differentiated odontoblasts and the HERS zone (Fig. 1a). USP34 positive cells were also scattered in the pulp tissue of mice (Fig. 1a). Similar result was observed in human dental pulp tissues, which showed wide distribution of USP34 in odontoblasts and pulp cells (Fig. 1b). In addition, we isolated the primary human DPCs and induced toward odontoblast differentiation. An increased expression of USP34 was observed at both mRNA and protein levels (Fig. 1c, d).

Next, we knocked down *USP34* in DPCs via siRNA (Fig. S1a, b). Depeletion of *USP34* inhibited the ALP activity and ARS staining (Fig. S1c). qRT-PCR revealed that the mRNA levels of related markers were decreased in *USP34* knockdown group (Fig. S1e). In addition, there was no significantly change in cell proliferation measured by EdU incorporation (Fig. S1f).

### Ablation of *Usp34* results in short root anomaly

**Fig. 2:**
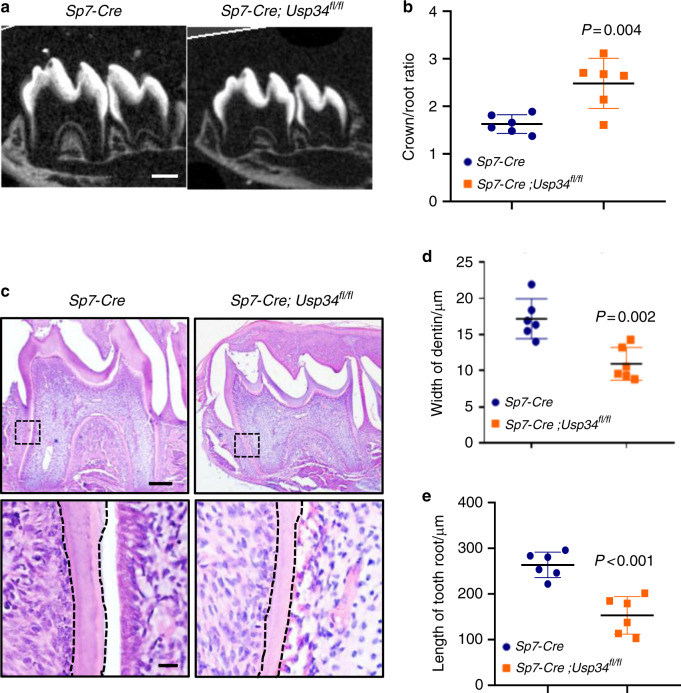
Conditional deletion of *Usp34* in the dental mesenchyme cells results in short root anomaly. **a** Representative μCT images of the mandibular first molars from P14 littermate *Sp7-Cre* control and *Sp7-Cre;Usp34*^*fl/fl*^ mice. Scale bar: 50 μm. **b** Quantification of the crown/root ratio of the first mandibular molars from μCT (*n* = 6). **c** Hematoxylin and Eosin staining of the mandibular first molar sections. Boxed areas are shown at higher magnification. Scale bar: 100 μm and 10 μm (boxed areas). **d**, **e** Quantification of dentin width and root length of the mandibular first molars (*n* = 6)

Next, we generated *Usp34* specific knockout mice through conditional cre expression driven by the osterix promoter.^23^ *Sp7-Cre;Usp34*^*fl/fl*^ mice were viable and born at the expected Mendelian ratio. We then performed μCT analysis of the first mandibular molars and showed that the growth of roots in *Sp7-Cre;Usp34*^*fl/fl*^ mice was apparently stunted at the age of 2 weeks (Fig. 2a, b). The root length and dentin thickness of *Sp7-Cre;Usp34*^*fl/fl*^ mice were significantly reduced, manifested by a phenotype with short roots and thin root canal walls (Fig. 2a, b). This change was also confirmed by histomorphometric analysis (Fig. 2c–e).

### USP34 regulates the protein level of NFIC

**Fig. 3:**
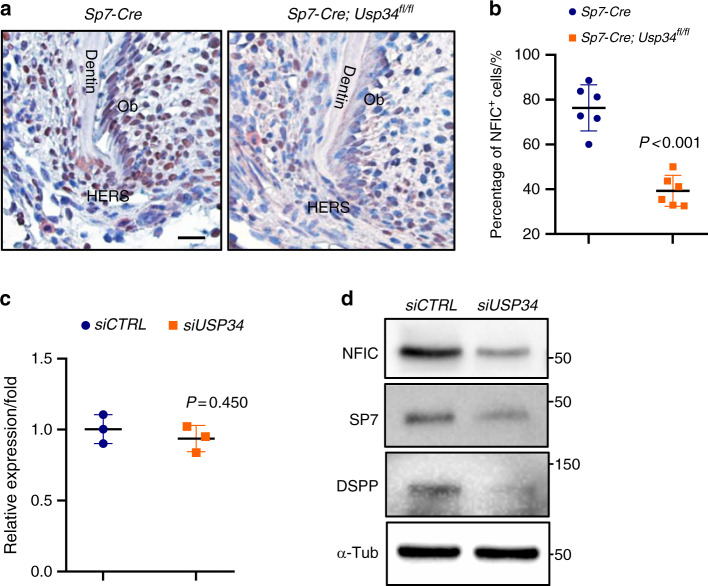
Loss of USP34 results in decreased expression of NFIC. **a** Immunohistochemistry of NFIC in the HERS region of mandibular first molars from littermate *Sp7-Cre* control and *Sp7-Cre;Usp34*^*fl/fl*^ mice. Scale bar: 10 μm. **b** Quantification of NFIC positive cells ratios (*n* = 6). Ob Odontoblasts. **c** qRT-PCR analysis of *NFIC* in *USP34*-deficient DPCs. No difference was observed (*n* = 3). **d** Western blot analyses showed decreased protein levels of NFIC, SP7, and DSPP

To unveil the molecular change of this phenotype, we examined several proteins which are involved in tooth development and regulated by ubiquitin. Interestingly, we found that NFIC, a vital regulator of root formation, reduced dramatically after *Usp34* knockout. Immunohistochemistry of NFIC showed the decreasing tendency of odontoblasts in apical zone in P14 molars from *Sp7-Cre;Usp34*^*fl/fl*^ mice as compared with the littermate controls (Fig. 3a). Quantitative analysis of the NFIC^+^/DAPI^+^ nuclei rate verified this observation (Fig. 3b). Western blot showed a significant reduction of protein level of NFIC in *USP34*-deficient DPCs, while the mRNA level remained unchanged (Fig. 3c, d). Depletion of *USP34* in DPCs also led to decreased expression of SP7 and DSPP, the downstream targets of NFIC (Fig. 3d).

### USP34 deubiquitinates and stabilizes NFIC

**Fig. 4:**
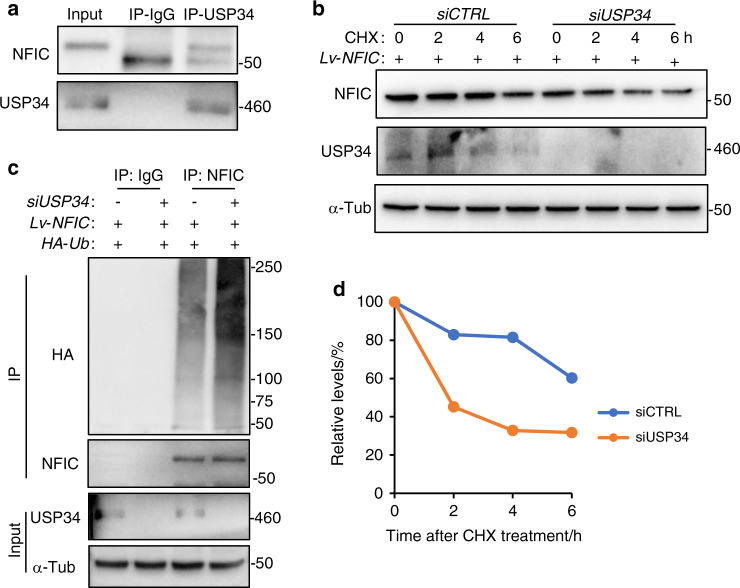
USP34 deubiquitinates and stabilizes NFIC. **a** Co-immunoprecipitation of USP34 with ectopically expressed NFIC in 293 T cells. **b** Loss of USP34 increased the degradation rate of NFIC as determined by CHX chase assay. 293 T cells were starved overnight, treated with CHX and harvested at indicated time periods. **c** Immunoblot of NFIC-linked polyubiquitin. 293 T cells were transfected with *siUSP34*, *HA-Ub*, and then treated with 10 μM MG132 for 4 h. **d** Quantification of NFIC degradation rate

To uncover the molecular interaction between USP34 and NFIC, we transduced 293 T cells with *Lv-NFIC* and then performed co-IP analysis. NFIC was successfully detected in USP34 immunoprecipitates (Fig. 4a). Ubiquitination assay showed increased ubiquitination level of NFIC in *USP34*-deficient 293 T cells (Fig. 4c), indicating the presence of USP34-dependent deubiquitination of NFIC. Furthermore, we treated 293 T cells with cycloheximide (CHX), a protein translation inhibitor, and observed that depletion of *USP34* accelerated the degradation of NFIC (Fig. 4b, d).

### Overexpression of *NFIC* partially rescues the odontogenic potential of *USP34*-deficient DPCs

**Fig. 5:**
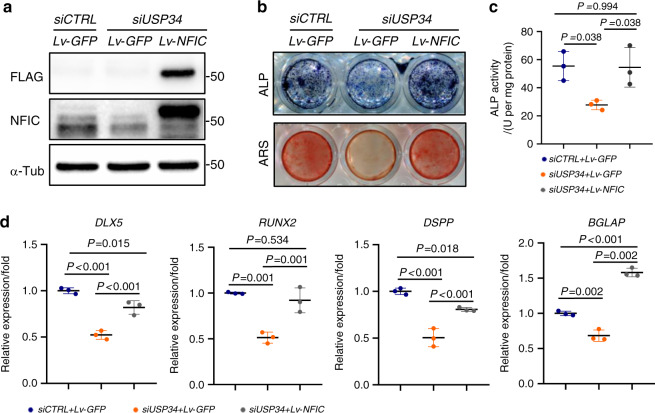
Overexpression of NFIC partially restores the odontogenic potential of USP34-deficient DPCs. **a** Western blot analysis of NFIC expression in DPCs. **b** Representative images of ALP and ARS staining for DPCs cultured in differentiation medium for 10 days and 21 days, respectively (*n* = 3). **c** Quantification of ALP activity (*n* = 3). **d** qRT-PCR analysis of the expression of odontogenic markers in DPCs (*n* = 3)

To test whether USP34-dependent deubiquitination of NFIC is involved in odontogenic differentiation of DPCs, DPCs were infected with *Lv-NFIC*, or *Lv-GFP* as control. *Lv-NFIC* successfully restored the protein level of NFIC in *USP34*-depleted DPCs (Fig. 5a), and partially rescued their odontogenic capacity, as shown by the upregulation of ALP activity and calcium mineralization (Fig. 5b, c). This observation was further confirmed by the restoration of related odontogenic gene expression, including *DLX5*, *RUNX2*, *DSPP*, and *BGLAP* (Fig. 5d).

## Discussion

The root is an indispensable structure of tooth when performing biological function. There are numerous factors reported to affect the root structure, such as dental trauma, periodontal diseases, and hereditary syndromes. Given the urgency of efficient therapeutic methods for root defects in clinic, tooth regeneration has been presented as a promising therapeutic target.^26–28^ Figuring out the molecular mechanisms of root morphogenesis is an inevitable step for developing new therapeutic approaches.^29^ Reducing expression of Gli1 by suppressing hedgehog can partially restore the proliferation ability of DPCs, promoting root elongate in Nfic-deficient mice.^30^ Depletion of β-catenin can reverse the abnormal differentiation trend of crown epithelia causing by BMPr1a ablation.^31^ Here we firstly report that USP34, an important deubiquitinase, controls root morphogenesis via stabilizing NFIC.

The activation of USP34-induced deubiquitination was proved to be present frequently in numerous diseases, including osteoporosis, cranio-facial dysmorphisms, and prostate cancer.^23,32,33^ There are experiments using *Usp34*-conditional knockout mice to investigate its tissue-specific roles during development. For example, *Prx1-Cre;Usp34*^*fl/fl*^ mice knocked out *Usp34* in mesenchymal stem cells and displayed severe skeletal defects.^23^ Dental mesenchymal cells adhering to HERS are essential for downgrowth of roots and dentin formation. In the present study, we observed short root anomaly after deletion of *Usp34* in *Sp7* positive dental mesenchymal cells. There was also an apparent decrease of odontogenic differentiation and impairment of mineralization ability in *USP34-*deficient DPCs.

NFIC has been considered as a master gene in tooth root elongation,^34^ and *Nfic* knockout mice fail to form normal root of tooth.^30,35,36^ Remarkably, the deletion of *Nfic* had no effect on crown morphogenesis, confirming that the root and the crown have quite different developmental sequences controlled by different signaling molecules.^37^ Therefore, recent researches have been focusing on the intense hunt for signaling pathways affecting function of NFIC during tooth development.^30,38^ Previous study observed a complicated interaction between TGF-β signaling and NFIC via Smurf1-induced polyubiquitination during odontoblast differentiation.^20,21^ Our research identified that USP34 deubiquitinates and stabilizes NFIC to promote odontoblast differentiation and thus regulates root formation.

In addition, USP34 has also been reported to be involved in the ubiquitination modification of proteins in different signaling pathways, including Wnt/β-catenin and TGF-β signaling.^23,39^ Bmp signaling is found activated when dental mesenchymal cells start differentiating into odontoblasts, which initiates apical growth of root.^40,41^ It has also been confirmed that epithelial Wnt10a controls root furcation formation and regulates proliferation of adjacent mesenchymal cells.^10^ Upregulation of Wnt signaling by ablation of Runx2 would disturb normal odontoblastic differentiation.^12^ Considering the potential roles of these proteins in root formation, further studies will be required to investigate the coordination between them.

## Conclusion

In summary, we showed that USP34 regulates root morphogenesis and promotes dental mesenchymal cells to undergo odontogenic differentiation through deubiquitination and stabilizes NFIC. This discovery provides new insights into the biological function of deubiquitinating enzymes.

## Materials and methods

### Mice

*Usp34* conditional knockout mice in a background of C57BL6/J were generated by CRISP/Cas9-based approach as previously reported.^23^ *Sp7-Cre* transgenic mice were acquired from the Jackson Laboratory (Bar Harbor, ME) and mated with *Usp34*^*fl/fl*^ mice to finally obtain *Sp7-Cre;Usp34*^*fl/fl*^ conditional knockout mice. The littermate *Sp7-Cre* mice were used as the control group.

Purified genomic DNA extracted from mice tails was used for PCR-based genotyping. Mice were housed and bred in a specific pathogen-free condition with an illumination cycle of 12-h light and 12-h dark. All procedures were conducted in compliance with the protocols approved by the Subcommittee on Research and Animal Care (SRAC) of Sichuan University.

### Micro-computed tomography (μCT) analysis

Mandibles including molars and incisors were harvested and then fixed in 4% paraformaldehyde (PFA) at 4 °C for 24 to 48 h. Samples were stored in PBS (pH 7.4) at 4 °C before being processed. The mandibles were scanned at a spatial resolution of 5 μm and a medium resolution using a μCT 50 microCT system (Scanco Medical, Bassersdorf, Switzerland). The 3D images of the first molars were reconstructed from the scan slice data.

### Tissue processing and histological staining

The dissected mandibles were fixed in 4% PFA at 4 °C for 24 to 48 h, and then decalcified in 10% ethylenediaminetetraacetic acid (EDTA) for 3 to 4 weeks. After being dehydrated through a series of graded ethanol, samples were carefully embedded in paraffin to ensure the parallelism of sectional plane and then were cut into five-μm-thick sections using a microtome (Leica RM2235). For hematoxylin-eosin (HE) staining, sections were deparaffinised in xylene and rehydrated in graded ethanol. Sections were stained with hematoxylin for 10 s, followed by eosin for 15 s. After staining, sections were dehydrated through 100% ethanol and cleared in xylene.

### Immunohistochemistry

Sections were deparaffinised in xylene and rehydrated in graded ethanol. To reduce endogenous peroxidase activity, sections were treated with 3% hydrogen peroxide for 15 min. Then slices were immersed in sodium citrate buffer for 15 min at 99 °C for antigen retrieval and blocked with 5% BSA for 30 min afterwards. The primary antibodies, rabbit anti-USP34 (1:100; A300-824A; Bethyl) or rabbit NFIC antibody (1:200; 16399-1-AP; Proteintech), were incubated overnight at 4 °C. Goat anti-rabbit IgG secondary antibody was applied for 1 h the following day and then the sections were detected by AEC Staining Kit (Boster Biological Technology).

### Cell isolation and culture

To obtain human DPCs, we collected young permanent molars with healthy dental pulp tissues after obtaining informed consent, complying with the informed protocol approved by the Committee on Human Research of the West China Hospital of Stomatology of Sichuan University. The dental pulp tissues were isolated from teeth and shred into tissue fractions. Then we incubated the tissue fractions with 3% Collagenase I (Sigma) at 37 °C for 1 h. After that, the digested tissues were transferred to α-minimum essential medium (α-MEM) (Hyclone Laboratories) containing 10% fetal bovine serum (FBS, Gibco), 100 U·mL^−1^ penicillin, and 100 μg·mL^−1^ streptomycin (Gibco) at 37 °C with a humidified atmosphere of 5% CO_2_. Adherent cells were collected and further cultured in odontogenic medium, serum-containing α-MEM supplemented with 5 mmol·L^−1^ β-glycerophosphate (Sigma), 50 μg·mL^−1^ ascorbic acid (Sigma), and 100 nmol·L^−1^ dexamethasone (Sigma).

For small interfering RNA (siRNA) experiments, cells were transfected with *USP34 siRNA* (Santa Cruz Biotechnology) with the recommended amounts of Lipofectamine RNAiMax (Invitrogen). The efficiency of the gene knockdown was assessed.

For NFIC overexpression experiments, lentiviruses expressing empty vectors GFP and NFIC were purchased from Genechem (Shanghai, China). Cells were cultured and then infected with a lentivirus at a MOI = 10. Afterwards, cells were treated with 2 μg·mL^−1^ puromycin (Sigma) for selection, and used in subsequent experiments.

### EdU (5-ethynyl-2-deoxyuridine) cell proliferation assay

Cells on cover glass were incubated with odontogenic medium containing 50 μmol·L^−1^ EdU for 8 h and were fixed with 4% PFA for 20 min. EdU was labeled with Click-iT EdU Imaging Kit (Invitrogen). Then sections were further incubated with DAPI to strain nuclei and imaged by laser scanning confocal microscopy (LSCM; Olympus FV3000).

### Alkaline phosphatase (ALP) and mineralization assays

Cells were cultured in odontogenic medium for 10 days and fixed with 4% PFA. After washing with PBS, cells were stained with 0.1 mol·L^−1^ Tris buffer (pH 9.3) containing 0.75% Fast Blue (Sigma) and 0.25% naphthol AS-BI phosphate (Sigma). The quantification of ALP activity was assayed with a commercial kit (Cell Biolab) and determined by absorbance measurements at 450 nm using a spectrophotometer (Thermo Fisher Scientific).

For alizarin red staining (ARS), cells were cultured and induced into odontoblasts for 21 days. Fixed with 4% PFA, the cells were then stained with 2% Alizarin red S (pH 4.2, Sigma) for 30 min with gentle shaking.

### Quantitative real-time reverse transcription polymerase chain reaction (qRT-PCR)

Total RNA from DPCs was extracted with Trizol Reagent (Invitrogen) according to the manufacturer’s instructions. cDNA was prepared with 1 μg total RNA using a reverse transcript kit (Takara). qRT-PCR was performed using the CFX96 Real-Time System (Bio-Rad Laboratories) with SYBR Premix Ex Taq II (Takara). Relative gene expression was analyzed using a 2^-ΔΔCt^ method and normalized to the expression of the housekeeping gene *Gapdh*.

### Western blot

Total protein was obtained using radioimmunoprecipitation assay (RIPA) buffer (Pierce) on ice. After centrifugation at 13 000 *g* for 15 min at 4 °C, the lysate was heated at 95 °C for 5 min in SDS loading buffer. The samples were then separated on 6% or 10% SDS-polyacrylamide gels and transferred to polyvinylidene difluoride (PVDF) membranes by a wet transfer apparatus (Bio-Rad). The membranes were blocked with 5% skim milk powder at room temperature for 1 h and incubated with primary antibodies overnight at 4 °C. The primary antibodies used include: rabbit anti-USP34 (1:2 000; A300-824A; Bethyl Laboratories), rabbit NFIC antibody (1:3 000; 16399-1-AP; Proteintech), mouse OSX antibody (1:100; sc-393325; Santa Cruz Biotechnology), mouse DSPP antibody (1:100; sc-73632; Santa Cruz Biotechnology), rabbit α-tubulin antibody (1:5 000; 11224-1-AP; Proteintech), mouse FLAG antibody (A8592; Sigma), rabbit anti-HA (1:1 000; 3724; Cell Signaling Technology). The PVDF membranes were then incubated with horseradish peroxidase conjugated anti-rabbit or anti-mouse IgG secondary antibodies (Cell Signaling Technology) and visualized using Immobilon reagents (Millipore).

### Co-immunoprecipitation (Co-IP)

293 T cells were transfected with lentiviruses expressing *NFIC* (Genechem) and then lysed in mild lysis buffer containing protease/phosphatase inhibitor cocktail (5872 S; Cell Signaling Technology) for 10 min with gentle shaking and then centrifuged at 14 000 *g* for 10 min at 4 °C. About 10% of the supernatant was harvested as inputs, and the remaining cell lysate was incubated with designed antibodies at 4 °C overnight with mixing. Protein A/G magnetic beads (TJ273976A; Thermo Fisher Scientific) were then added and incubated for another 1 h at 4 °C. The beads were collected and washed with mild lysis buffer, followed by Western blot analysis.

### Ubiquitination assay

We constructed lentivirus to overexpress *NFIC* (*Lv-NFIC*) or *GFP* (*Lv-GFP*) as a control in 293 T cells and treated cells with 2 μg·mL^−1^ puromycin to select. 293 T cells were then transfected with HA-Ubiquitin (17608; Addgene) and *USP34 siRNA*. Cells were treated with 10 μmol·L^−1^ MG132 for 4 h before collection of cell lysates. The supernatant was co-immunoprecipitated with anti-NFIC antibody and protein A/G magnetic beads. Then the sample was subjected to Western blot analysis.

### Statistical analysis

All values are expressed as mean ± SD. Statistically significant differences were performed by two-tailed Student’s *t* test for comparison between two groups or by one-way analysis of variance (ANOVA) followed by the Tukey’s post hoc test for multiple comparisons. A *P*-value of less than 0.05 was considered to be statistically significant.

## Supplementary information

Supplementary Information
