# Supplementary material for: USP34 regulates tooth root morphogenesis by stabilizing NFIC
Source: Int J Oral Sci. 2021 Mar 9;13:7. doi: 10.1038/s41368-021-00114-8 (PMC7940473; doi:10.1038/s41368-021-00114-8)
Supplement: Supplementary file 1 — Supplementary Information [file 41368_2021_114_MOESM1_ESM.docx]

**Supplementary figure S1**

**
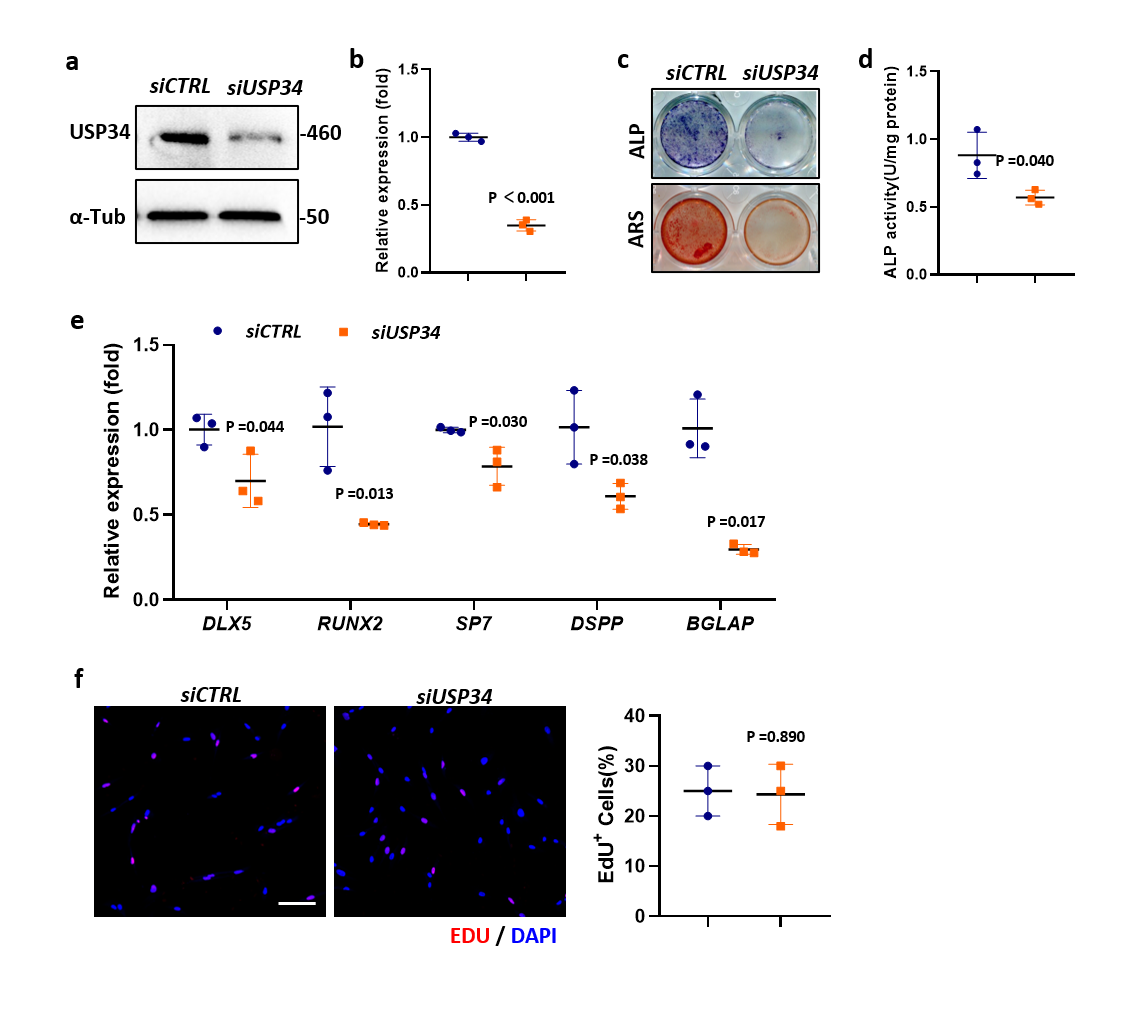
**

**Figure S1. Depletion of *USP34* impairs odontogenic differentiation of DPCs.** (a, b) The knockdown efficiency of *USP34* in the DPCs was confirmed by western blot and qRT-PCR (n=3). (c) Representative images of ALP and ARS staining for *USP34*-deficient DPCs (n=3). (d) Quantification of ALP activity (n=3). (e) qRT-PCR detected the mRNA expression change of odontogenic markers in *USP34*-knockout DPCs (n=3). (f) Representative images and quantitative analyses of cell immunofluorescence staining of EdU (n=3). Scale bar: 50μm.
